# Chitosan-Entrapped TiO_2_ Nanoparticles Synthesized Using *Calendula officinalis* Flower Extract—Photophysical Characterization, Biocompatibility, and Textile Dye Remediation

**DOI:** 10.3390/polym18060745

**Published:** 2026-03-19

**Authors:** Sushmitha Sundarraj, Sridhanya Mysore Shreethar, Nivitha Shri Chandrasekaran, Koyeli Girigoswami

**Affiliations:** 1 Saveetha Medical College and Hospital, Saveetha Institute of Medical and Technical Sciences, Thandalam, Chennai 602105, India; 2 Medical Bionanotechnology Laboratory, Department of Obstetrics and Gynaecology, Saveetha Medical College and Hospital, Saveetha Institute of Medical and Technical Sciences, Thandalam, Chennai 602105, India

**Keywords:** remediation, *Calendula officinalis*, crystal violet, zebrafish embryos, titanium dioxide

## Abstract

Effluents from industries, manufacturing companies, textile looms, and floodwater contaminate the surface water reservoirs. This endangers the quality of water for use by humans. Wastewater remediation is one of the ways to recycle the dirty water and make it suitable for use. Photocatalysis is the most common method for wastewater remediation, especially using Titanium dioxide (TiO_2_) nanoparticles. However, chemical synthesis and direct addition of nanoparticles may cause toxicity to the flora and fauna present in the water body. To address this limitation, we have green-synthesized TiO_2_ nanoparticles using a horticulture waste, *Calendula officinalis* dried flower extract and entrapped them in a natural polymer, chitosan (CTS-TiO_2_-CO nanocomposite). The polymer entrapment ensures biocompatibility as well as reduced aggregation of nanoparticles. The synthesized CTS-TiO_2_-CO nanocomposite was characterized using UV-visible spectrophotometry, dynamic light scattering, zeta potential, Fourier Transformed Infrared Spectroscopy (FTIR), X-ray diffractometry (XRD), scanning electron microscopy (SEM) and energy-dispersive X-ray spectroscopy (EDAX) analysis. The absorption peak was found at 302 nm, and the hydrodynamic diameter at 490 nm. SEM images show flower-like morphology with 326 nm average particle diameter. The non-toxic dose of the nanoparticles was estimated by MTT assay and zebrafish embryo developmental studies. More than 82% fibroblast cells were viable after treatment with 100 μg/mL of CTS-TiO_2_-CO nanocomposite. 85% embryos hatched after treatment with 50 μg/mL of CTS-TiO_2_-CO nanocomposite. Further, the textile dye remediation assessment was done using the dye crystal violet, exhibiting 69.19% dye degradation after 4 h of sunlight exposure. Altogether, the results demonstrate that the CTS-TiO_2_-CO nanocomposite was effective in the remediation of crystal violet without causing any toxicity up to a dose of 100 μg/mL.

## 1. Introduction

The accelerated growth of the textile and dye industries has led to the increased discharge of synthetic dyes and chemical pollutants into the aquatic ecosystems. These contaminants, mainly azo dyes, are visually unappealing and create serious health risks and environmental pollution due to their toxicity, resistance to degradation and carcinogenicity [1]. Wastewater remediation is the process used to remove contaminants from water to make it safe to reuse for environmental discharge [2,3]. Conventional methods for wastewater treatment, including biological oxidation, flocculation, and coagulation, are partially effective in removing persistent organic pollutants, mainly from large-scale industrial effluents [4]. Moreover, harsh chemicals like bleach and chlorine-based compounds are used during the remediation process. However, these methods also pose a threat to the biological activities of the organisms dwelling in the water bodies to be remediated. There can be death of certain labile organisms and plants, damage to their DNA, or resistance can also be induced towards these harsh chemicals, which can be detrimental in the long run. Photocatalysis has gained popularity as a promising and sustainable technique for the remediation of wastewater [5]. Using photocatalysts also poses certain challenges, like aggregation, inability to recover the photocatalyst when added to large water bodies, and limited performance when used in over-polluted water.

Nanotechnology has become a transformative field with applications in environmental [6], biomedical [7], and energy sectors [8]. Metal and metal oxide nanoparticles have been extensively studied for their excellent properties at the nano scale due to the quantum confinement effect and huge surface area to volume ratio [9,10]. Among diverse metal oxide nanoparticles, titanium dioxide (TiO_2_) nanoparticles have gained excellent attention due to their excellent photocatalytic activity, non-toxic nature, and chemical stability [11]. However, the absorption of light in the UV range and rapid electron-hole combination have limited the use of nano-TiO_2_ for photocatalysis under visible light. Metal and non-metal doping, heterostructure development, and dye sensitization improve the activity of TiO_2_ in the visible region [12]. To overcome these shortcomings, modification of TiO_2_ was done, which includes tetragonal BaTiO_3_ nanoparticles, engineered hetero-catalyst BaTiO_3_/ZnO, pH-induced ZnO-TiO_2_ multi-phase composite, heterojunction of BaTiO_3_/g-C_3_N_4_, composites of TiO_2_-zeolite, etc. [13]. The TiO_2_ integration with bismuth-based photocatalysts can also improve the photocatalytic activity in the visible region. A few such composites include BiVO_4_, Bi_2_MoO_6_, and Bi_2_WO_6_ [14]. *Mentha aquatica* leaves were used to synthesize TiO_2_ nanoparticles that were decorated with activated carbon for the remediation of organic dyes [15]. Aggregation of TiO_2_ also creates issues, which can be addressed by synthesizing biopolymer-supported photocatalysts utilizing cost-effective, biodegradable and eco-friendly biopolymers, like chitosan, cellulose, alginate, cyclodextrin, starch, and guar gum [16].

Conventional synthesis methods involve hazardous chemicals, increased energy consumption, and low biocompatibility [17,18,19,20], which prompts the green synthesis exploration since it is both eco-friendly and cost-effective [21,22,23]. Rather than using a conventional method, the use of plant extracts as a reducing and stabilizing agent in the synthesis of nanoparticles offers a sustainable alternative [23]. *Calendula officinalis* belongs to a type of marigold that has many medicinal applications in virtue of containing some vital phytochemicals like flavonoids, phenolic acids, and terpenoids, which can exert antimicrobial, antifungal, anti-inflammatory, and antioxidant activities [24,25,26,27,28,29,30,31,32]. These phytochemicals facilitate the reduction in metal ions and nanoparticles’ stabilization, which eliminates the need for synthetic agents. In a study by Zhao et al. TiO_2_ nanoparticles were synthesized using *Calendula officinalis* flower aqueous extract and were used to reduce 4-nitrophenol to suppress conjunctivitis [33].

Biocompatibility and sustained release to the environment remain an unresolved problem for the nanoparticles [34,35]. Once the nanoparticles are entrapped inside a biologically safe polymer, they can be released to the environment for a long time. Moreover, they also become benign in nature. Chitosan, a natural polysaccharide derived from chitin, has special properties such as biodegradability, biocompatibility, and thin film-forming ability, making it unique for nanoparticle entrapment. Chitosan is not only used for stabilizing nanoparticles, but also improves their functional properties by providing active surface groups for interaction [36,37,38]. Thus, entrapping the green-synthesized TiO_2_ nanoparticles inside chitosan ensures slow and sustained release. This can exert its effect for a long time and does not cause any damage to living beings due to a burst release in the environment.

The current study aims to synthesize chitosan-entrapped TiO_2_ nanoparticles via a green synthesis approach using *Calendula officinalis* flower extract and to investigate their morphological, structural, and optical properties. The synthesized nanoparticles were subjected to many analytical techniques: phytochemical analysis and gas chromatography-mass synthesis (GC-MS) were done to identify the active photo constituents present in the *Calendula officinalis* flower extract, involved in the synthesis, Fourier transform infrared spectroscopy (FTIR) identified functional groups and bonding interactions, X-ray diffraction (XRD) confirms the crystallinity, zeta potential analysis and dynamic light scattering (DLS) provides insights into particle size and stability. Morphological and elemental characterization were conducted using Scanning electron microscopy (SEM) and energy dispersive X-ray spectroscopy (EDX), respectively. Further, the biocompatibility of the nanoparticle was studied using MTT assay, in vitro and zebrafish embryos, in vivo. Finally, the ability of the synthesized nanoparticle towards textile dye degradation was tested using the dye crystal violet.

## 2. Materials and Methods

### 2.1. Materials

The materials purchased for the study were all analytical grade. Isopropanol (99.8%), glacial acetic acid (99.5%), titanium tetraisopropoxide, Dulbecco’s minimal essential medium, antibiotic-antimycotic solution (penicillin, streptomycin, and amphotericin B), MTT, dimethyl sulphoxide (DMSO), chitosan, and crystal violet (C.I. 42555) were purchased from HiMedia Pvt. Ltd., Mumbai, Maharashtra, India. Gibco, Life Technologies Corporation, Grand Island, NY, USA, supplied the Fetal bovine serum. Other chemicals were obtained locally. NCCS, Pune, Maharashtra, India, was the source of cell lines, and zebrafishes were obtained from a local authorized vendor, Tarun Fish farm, 2/32C, East Mada Street, Manimangalam, Chennai, India. Every glassware was washed using aqua regia and rinsed with tap water, followed by distilled water, before beginning the experiments.

### 2.2. Preparation of Calendula Flower Extract and Analysis

*Calendula officinalis* (CO) flowers were purchased from Amazon India Ltd. (Bengaluru, India). The powder was washed completely with distilled water, and surface contaminants such as soil and dust particles were eliminated. 0.25 g of the powder was taken in 100 mL of distilled water and heated up to 70 °C with continuous stirring for 2 h using a magnetic stirrer. Then the extraction was continued using ultrasonication for 15 min at 40 kHz frequency, four times. Finally, Whatman filter paper was used to filter the extract and kept at 4 °C for further use.

Phytochemical analysis of the *Calendula officinalis* flower extract was investigated to find the phytochemicals present in it according to Garg P et al. 2018 [39]. The respective tests were performed for the identification of saponins, tannins, terpenoids, alkaloids, flavonoids, and carbohydrates, respectively, as done previously [40,41].

GC-MS analysis (Perkin Elmer Clarus 680 and 600, Perkin Elmer, Shelton, CT, USA) was carried out to identify the chemical components present in the *Calendula officinalis* flower extract according to Jannai et al. [42]. The aqueous extract of *Calendula officinalis* flowers was completely evaporated using a rotary evaporator (Aditya Scientific, Hyderabad, Telangana, India) and resuspended using ethanol, and then subjected to GC-MS analysis. The compounds were identified based on their retention time, fragmentation pattern, and through NIST-based automated mass spectral deconvolution and identification software.

### 2.3. Green Synthesis of TiO_2_ Nanoparticles via Sol–Gel Method

Sol–gel method was used to synthesize the TiO_2_ nanoparticles [43]. Under vigorous magnetic stirring, isopropanol (5.2 mL) and glacial acetic acid (2.5 mL) were mixed for 15 min by covering the beaker with an aluminum foil to avoid evaporation. 2.5 mL of titanium tetraisopropoxide (TTIP) was added dropwise slowly into the solvent mixture under vigorous magnetic stirring and mixed for 30 min. 1.7 mL of the prepared *Calendula officinalis* flower extract was added for the gel formation, and the stirring was continued till a pale-yellow gel was formed. Then the gel was dried in a hot air oven at 240 °C to form a dry lumped powder. Using a mortar and pestle, the product was ground into a fine powder. The resulting product was calcined at 300 °C for 2 h to obtain crystalline CO-TiO_2_ nanoparticles.

### 2.4. Preparation of Chitosan Solution

**Figure 1:**
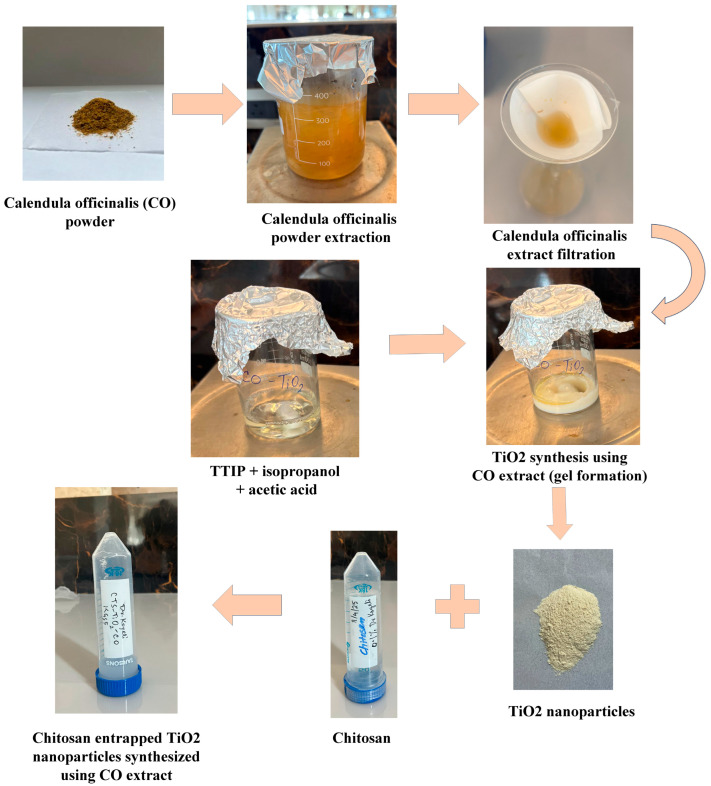
The steps of synthesis of CTS-TiO_2_-CO nanocomposite. At first, the *Calendula officinalis* aqueous extract was prepared and filtered. Then, the TiO_2_ nanoparticles were synthesized using the CO extract by the sol–gel method. Then, a chitosan solution was prepared, and TiO_2_ nanoparticles were mixed with the chitosan solution and sonicated using an ultrasonication water bath. This gives the final product chitosan-entrapped TiO_2_ nanoparticles synthesized using CO extract (CTS-TiO_2_-CO nanocomposite).

Chitosan (CTS) solution was prepared by dissolving 0.5 g of chitosan in 50 mL of 1% glacial acetic acid. This mixture is stirred using a magnetic stirrer for 2 h until a clear homogeneous solution is obtained. This chitosan solution was used for synthesis or after TiO_2_ nanoparticle formation to coat or entrap the particles for enhanced stabilization and biocompatibility. 0.025% chitosan was taken along with 10 mg of CO-TiO_2_ nanoparticles, and sonicated for 1 h (20 min × 3 times). This gives our final product CTS-TiO_2_-CO nanocomposite. The pH of the final solution was adjusted to 7.2. The steps of synthesis of CTS-TiO_2_-CO nanocomposite are shown in Figure 1.

### 2.5. Characterization of Nanoparticles

The nanoparticles were characterized using the methods done earlier [44,45]. Dynamic Light Scattering (DLS) provides the hydrodynamic diameter, and zeta potential provides information on the surface charge of the nanoparticles. UV-visible spectrophotometry provides the characteristic absorption spectrum of a nanoparticle. DLS and zeta potential were recorded using Malvern Zetasizer (Malvern Zetasizer Nano ZS, Malvern Panalytical Limited, Spectris, Grovewood Road, Malvern, UK). Absorption spectrum in the UV and visible range was recorded using a JASCO UV-VIS, V-730 double beam spectrophotometer, JASCO UK Limited, Yorkshire, UK. The CTS-TiO_2_-CO nanocomposite (50 μL) and CO-TiO_2_ nanoparticles were diluted using distilled water (2 mL), sonicated in an ultrasonicator water bath (LabQuest, Borosil Scientific Limited, Bandra (East), Mumbai, India) for proper dispersion and subjected to DLS, zeta potential and UV visible spectrum measurement. A regular polystyrene cuvette was used for hydrodynamic diameter measurement, and a quartz cuvette was used for UV-vis absorption. A disposable capillary cell was used to record the zeta potential. Tauc’s plot was plotted using the UV spectrum data, and the band gap energy was calculated. Sample preparation of SEM involves a thin layer coating of the CTS-TiO_2_-CO nanocomposite above a carbon tape and drying it in a dust-free atmosphere. Post drying, the samples were sputter coated with Au-Pd alloy to make them conductive and subjected to SEM analysis (FEI–TECNAI, G2-20 TWIN), Thermo Fisher (Thermo Fisher Scientific, Waltham, MA, USA). Particle shape and surface morphology were examined by SEM. EDX was performed along with SEM to analyze the elemental composition.

Sample preparation for XRD and FTIR involved heat drying of CTS-TiO_2_-CO nanocomposite at 70 °C to avoid charring of the polymer, chitosan. CO-TiO_2_ nanoparticles were used as such for the FTIR analysis. The complete dried sample was subjected to XRD analysis (Unique D8 diffractometer) using Cu Kα radiation (λ = 1.5406 Å) to determine the size and crystalline phases of the synthesized nanoparticles. The scan range is set between 10° and 80° (2θ). The functional groups of the samples were identified by Attenuated Total Reflectance–Fourier Transform infrared spectroscopy (Spectrum Two, Perkin Elmer, Shelton, CT, USA) in the spectral region of 4000–400 cm^−1^ wavelength with 4 cm^−1^ resolution at a total scan of 64 scans per sample.

### 2.6. In Vitro and In Vivo Biocompatibility Assessment

When we are applying any nanoparticle to the environment for remediation, we must estimate whether it is safe for the environment or not. To explore the safe dose of our synthesized nanoparticles, we estimated both in vitro and in vivo biocompatibility [42,44]. In vitro cell viability was assessed using normal Chinese hamster lung fibroblast cell lines, V79, after treatment with various doses of CTS-TiO_2_-CO nanocomposite, following the procedure done earlier [44]. 1 × 10^4^ cells were seeded in a 48-well plate and allowed to adhere for 24 h. Post adherence, treatment with nanoparticles was done (the samples were filtered using a syringe filter (0.22 μm) under sterile conditions and added to the wells). MTT solution (5 mg/mL) was made in DMEM and filtered using a 0.22 μm sterile syringe filter, and 100 μL was added to each well. The plates were allowed to stand in a CO_2_ incubator (CellXpert C170, Eppendorf India Private Limited, Ambattur, Chennai, India) for 4 h in the dark. The developed formazan crystals were solubilized using DMSO, and the measurement of absorbance was performed at 570 nm for cell viability calculation. In vivo biocompatibility was measured using the zebrafish (*Danio rerio*) embryo assay. The study was conducted according to the procedure followed earlier [46]. Briefly, the embryos at 8 h post fertilization (hpf) were exposed to the different doses of the CTS-TiO_2_-CO nanocomposite, and monitored under a light microscope at different time points for any abnormality. Each dose was done in triplicate, and in each well, 20 embryos were taken. The number of embryos hatched was noted, and cumulative hatchability was calculated. The animal study protocol was approved by the Institutional Animal Ethics Committee (IAEC approval No. SU/CLAR/RD/19/2025) dated 4 August 2025 from Saveetha Medical College and Hospital, SIMATS, for studies involving animals. The study was performed according to the OECD guidelines.

### 2.7. Textile Dye Remediation Study

Crystal violet was selected for our study because it is frequently used in staining for microbiology or molecular biology laboratories. It is also extensively used in the textile industry. A textile dye remediation study was done following the methodology used earlier [11]. The capability of the CTS-TiO_2_-CO nanocomposite to degrade crystal violet was assessed after exposure to different time intervals. Photocatalysis was the procedure employed for dye degradation. A 0.001% concentration of crystal violet dye was prepared in distilled water and divided into four tubes (5 mL in each tube). 100 μL of the synthesized CTS-TiO_2_-CO nanocomposite was added to each tube, and the tubes were exposed to sunlight for 0 h, 1 h, 2 h and 4 h. The percentage degradation of the dye was estimated using the formula given below:

Absorption maximum (λ_max_) of Crystal Violet is at 582 nm.


$$\%\;CV\;degradation=\frac{\left(OD\;at\;0\;h\right)-(OD\;at\;x\;h)}{\left(OD\;at\;0\;h\right)}\times 100$$

OD at 0 h indicated the absorbance of the sample immediately after the addition of the photocatalyst to the dye. In contrast, the OD at x h indicates the absorbance after 1 h, 2 h and 4 h exposure to sunlight.

### 2.8. Statistical Analysis

An unpaired Student’s *t*-test was used to calculate the statistical significance between the control and treated groups. *p* < 0.05 was considered to be significant compared to the untreated control group. Every experiment was conducted in triplicate, and the values are expressed as mean ± SEM.

## 3. Results and Discussion

### 3.1. Composition of Calendula officinalis Flower Extract

The phytochemical extracts obtained from *Calendula officinalis* were found to contain a diverse range of bioactive compounds, including saponins, tannins, carbohydrates, flavonoids, glycosides, reducing sugars, and coumarins (Figure S1a). These compounds contribute to the plant’s potential therapeutic properties. GC-MS analysis from the flower extract determined various chemicals present in the extract (Figure S1b). The respective peaks for the components are shown in Figure S1c–g, representing 2-Formylhistamine, Imidazole-5-carboxylic acid, 2-amino-, 4-tert-Butylphenol, TMS derivative, d-Mannitol, 1-decylsulfonyl-, and Oxiraneoctanoic acid, 3-octyl-, cis-, respectively. The chemical formula, molecular weight and abundance percentage are shown in Table S1. It is found that 2-Formylhistamine is 34.79% abundant in the CO extract, which can facilitate the capping of the nanoparticles and the bio-reduction of titanium ions.

### 3.2. Characterization of the Synthesized CTS-TiO_2_-CO Nanocomposite

**Figure 2:**
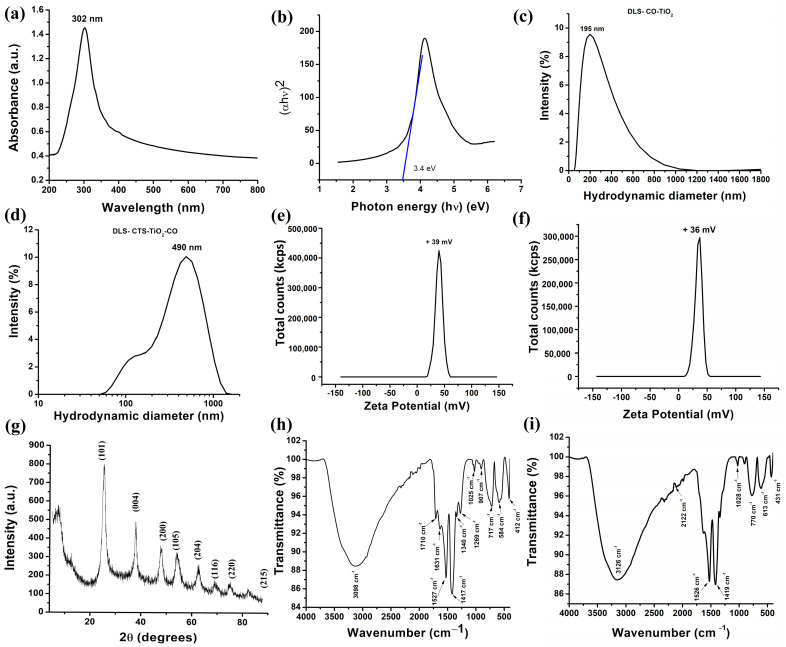
The various characterization results for the CO-TiO_2_ and CTS-TiO_2_-CO nanocomposite. (**a**) UV-visible spectrum (**b**), Tauc’s plot for band gap (**c**) hydrodynamic diameter for CO-TiO_2_ nanoparticles (**d**), hydrodynamic diameter of CTS-TiO_2_-CO nanocomposite (**e**) zeta potential of CO-TiO_2_ nanoparticles (**f**), zeta potential of CTS-TiO_2_-CO nanocomposite (**g**), X-ray diffraction spectrum of CTS-TiO_2_-CO nanocomposite (**h**) FTIR spectrum CO-TiO_2_ nanoparticles, and (**i**), FTIR spectrum of CTS-TiO_2_-CO nanocomposite.

The characteristic absorption spectrum of the synthesized CTS-TiO_2_-CO nanocomposite was measured from 200 nm to 800 nm. Figure 2a shows the absorption peak in the UV region at 302 nm, which is characteristic of a photocatalyst. In previous studies, it has been reported that TiO_2_ nanoparticles have an absorption peak at 286 nm due to the incorporation of impurities, showing the UV light-absorbing capacity of TiO_2_ nanoparticles [47]. Pure TiO_2_ nanoparticles, particularly in the anatase phase, are characterized by a fundamental absorption edge in the UV region (typically starting around 330–350 nm for bulk anatase and blue-shifting slightly for nanoparticles), not a discrete peak. This is due to the inter-band transition from the valence to the conduction band. The absorption profile should show a sharp, steep rise following the edge. The distinct absorption peak at 302 nm (Figure 2a) is uncharacteristic of the intrinsic band-gap absorption of TiO_2_. It is highly probable that this feature originated from the organic components of the composite, specifically from the π → π* or *n* → π* electronic transitions of the phytochemicals present in the *Calendula officinalis* extract (e.g., flavonoids, phenolic acids) or from the chitosan polymer. These organic molecules often exhibit strong, discrete absorption peaks in the UV region [48]. The band gap calculated using this UV data by Tauc’s plot was found to be 3.4 eV (Figure 2b). The bandgap energy decides the semiconducting nature of the synthesized nanoparticle, especially TiO_2_ [49,50]. A photocatalyst can absorb light energy, and it can initiate chemical reactions without being consumed itself. The major properties include an appropriate band gap (usually in the band gap range of a semiconductor), to absorb UV or visible light, possess a high surface area for effective reactant adsorption, and a high redox potential for the generation of reactive species such as hydroxyl radicals. The photocatalyst should be chemically and thermally stable under irradiation. They should have a low recombination rate of electron–hole pairs and should have good charge carrier mobility. In addition, a photocatalyst must be non-toxic up to the working doses, cost-effective, and reusable at least up to five cycles, making it appropriate for environmental remediation and energy conversion [51,52].

The hydrodynamic diameter gives an approximate estimation of the particle size. In this process, dynamic light scattering takes place in the diluted samples, which moves inside the liquid in a Brownian motion, and the particle is considered spherical in shape. There are layers of water molecules surrounding the particle, which also generates a potential difference between the inner and outer layers called the zeta potential. Zeta potential gives us the information on the stability of the nanoparticle in an aqueous medium. The more stable the nanoparticle, the less aggregation takes place [53,54]. The CO-TiO_2_ nanoparticles showed a hydrodynamic diameter of 195 nm (Figure 2c) and a zeta potential value of +39 mV (Figure 2e). On the other hand, for our synthesized CTS-TiO_2_-CO nanocomposite, we found the hydrodynamic diameter to be 490 nm (Figure 2d) and zeta potential as +36 mV (Figure 2f). The overall size of the chitosan-entrapped nanoparticles in suspension was 490 nm, which is larger than the core size of 190 nm, due to the hydration layer and polymer coating. The zeta potential value shows that our CO-TiO_2_ nanoparticles, as well as the CTS-TiO_2_-CO nanocomposite, were highly stable, because in magnitude, if the value of zeta potential is more than 25 mV, the particle is highly stable [54]. It has been shown earlier that polymer coatings, especially natural polymer, chitosan coating, improve the stability of metallic nanoparticles [55].

The XRD data of the dried samples of CTS-TiO_2_-CO nanocomposite (Figure 2g) showed the crystalline peaks corresponding to JCPDS-89-4921, which represent anatase TiO_2_ nanoparticles [56]. The peaks for hkl lattices found are—101, 004, 200, 105, 204, 116, 220, and 215, corresponding to the 2θ values of 25.220°, 37.915°, 47.730°, 54.208°, 62.650°, 68.932°, 74.821°, and 82.281°, respectively. The results corroborate the previous reports where authors have synthesized anatase TiO_2_ quantum dots for killing *E. coli* via photocatalysis [56]. Thus, the synthesis of our nanoparticles was crystalline in nature. The FTIR analysis of CO-TiO_2_ nanoparticles (Figure 2h) and CTS-TiO_2_-CO nanocomposite (Figure 2i) showed the following peaks (Table 1).

The FTIR peaks represent the different bond formations, which are necessary for the successful synthesis of our nanoparticle. FTIR peaks between 2800 and 3700 cm^−1^ represent O-H stretching, and the bands found at 1630–1660 cm^−1^ represent O-H-O scissors bending. Moreover, a weak band around 2122 cm^−1^ was found due to scissors bending with an enhanced liberation band near the IR region. The peaks present between 430 and 550 cm^−1^ are attributed to Ti-O-Ti vibrations, and the peaks at 770 cm^−1^ and 613 cm^−1^ represent Ti-O-Ti bending [61]. The corresponding peaks are present for only CO-TiO_2_, which was shifted to either higher or lower wavenumber, indicating the expansion or contraction of bonds to incorporate the TiO_2_ inside the chitosan matrix.

**Figure 3:**
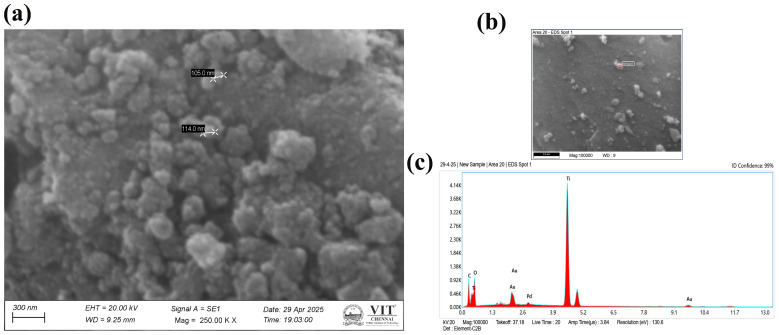
(**a**,**b**) The SEM image of CO-TiO_2_ nanoparticles at different magnifications. (**c**) The EDAX of the as-synthesized CO-TiO_2_ nanoparticles.

**Figure 4:**
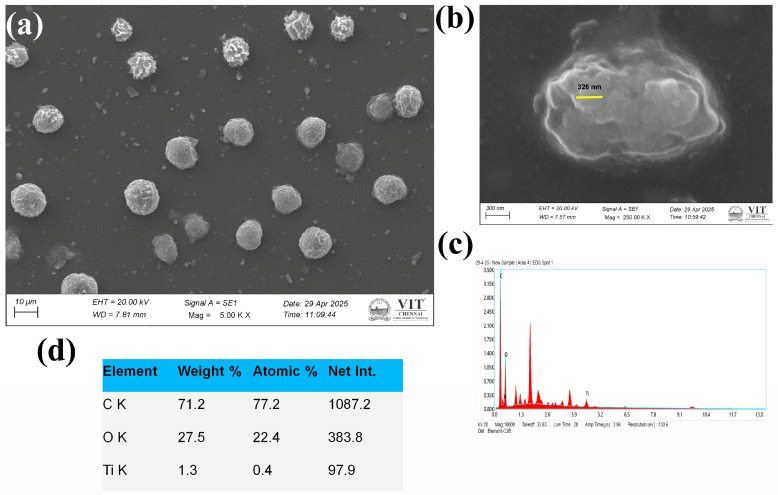
(**a**,**b**) The SEM image of CTS-TiO_2_-CO nanocomposite at different magnifications. (**c**) The EDAX of the as-synthesized CTS-TiO_2_-CO nanocomposite, (**d**) The elemental composition of CTS-TiO_2_-CO nanocomposite as analyzed by EDAX.

The SEM images of CO-TiO_2_ nanoparticles and CTS-TiO_2_-CO nanocomposite are shown in Figure 3 and Figure 4, respectively. The morphology of CO-TiO_2_ nanoparticles showed spherical structure with a diameter ranging from 105 to 114 nm (Figure 3a,b), whereas the CTS-TiO_2_-CO nanocomposites revealed peony flower-shaped spherical particles with a diameter of 326 nm (Figure 4a,b). The particle size is measured using the in-built software of the SEM instrument. However, some aggregation was also visible in the SEM image of CO-TiO_2_ nanoparticles. The TiO_2_ nanoparticles are moderately aggregated as visible in the SEM image of CTS-TiO_2_-CO nanocomposite, which is embedded in the chitosan matrix, giving their flower-like pattern. The EDAX analysis of both CO-TiO_2_ nanoparticles (Figure 3c) and CTS-TiO_2_-CO nanocomposite (Figure 4c) results confirms the presence of titanium (Ti) and oxygen (O), with traces of carbon (C), obtained from chitosan and plant biomolecules (Figure 4c). Additional peaks of Au and Pd can arise from the Gold-palladium coating done for SEM sample analysis (Figure 3c). The elemental composition of CTS-TiO_2_-CO nanocomposite is given in Figure 4d.

There was a difference in the size obtained by DLS and SEM for both CO-TiO_2_ nanoparticles and CTS-TiO_2_-CO nanocomposite, with DLS showing a higher diameter. The hydrodynamic diameter represents an estimated particle size because the instrument assumes that all particles are perfectly spherical. In the diluted suspension, the particles undergo Brownian motion within the dispersion medium, and the light they scatter is detected at a 90° angle. As the particles move through the medium, they are surrounded by a layer of associated water molecules, which increases the apparent measured size [53,54]. For this reason, the value is referred to as the hydrodynamic diameter rather than the true physical diameter. The actual particle size can be determined using transmission electron microscopy (TEM) or, to some extent, scanning electron microscopy (SEM). However, in SEM analysis, a thin coating of a gold–palladium alloy is typically applied to improve conductivity, which may slightly influence the measured diameter.

### 3.3. Biological Effect In Vitro and In Vivo

**Figure 5:**
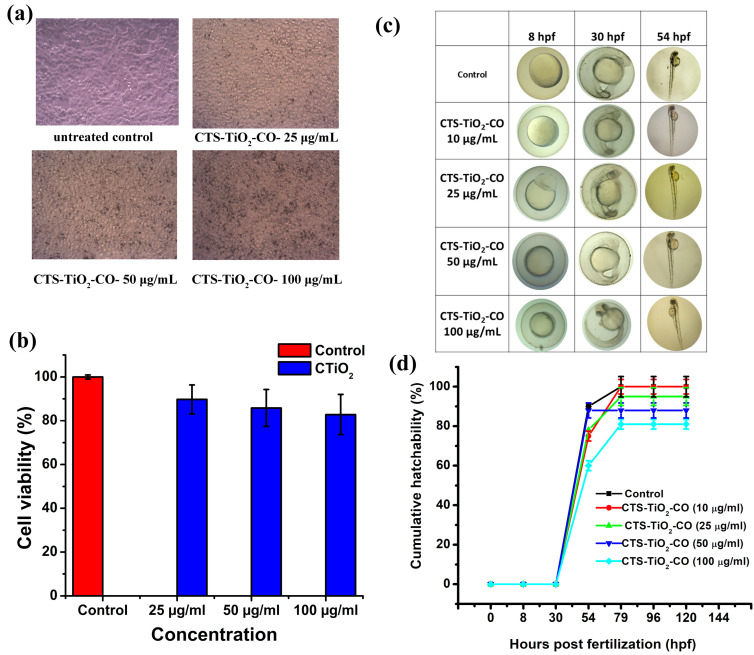
(**a**) The inverted microscopic images and (**b**) cell viability of V79 cells after treatment with different doses of CTS-TiO_2_-CO nanocomposite. (**c**) The microscopic images of zebrafish embryos captured after treatment with CTS-TiO_2_-CO nanocomposite and their (**d**) cumulative hatchability.

The biological effect of CTS-TiO_2_-CO nanocomposite was monitored in vitro as well as in vivo. This aspect becomes very important when we design a nanoparticle for environmental remediation use, since the nanoparticles will ultimately mix with the water body and can cause detrimental effects to the flora and fauna of the water body. The dose of exposure to any agent determines the toxicity of that agent, and hence, we should assess the safe dose that can be used for the remediation. Figure 5a shows the inverted microscopic images of normal fibroblast cells (V79, Chenise hamster lung fibroblasts) for untreated and 24 h treated with 25 μg/mL, 50 μg/mL and 100 μg/mL of CTS-TiO_2_-CO nanocomposite. The morphology of the cells appears normal and healthy, and there are no floating cells visible. This shows that the synthesized nanoparticles up to a dose of 100 μg/mL did not induce any cytotoxic changes in the cells. From Figure 5b, we could observe that the cell viability was 83 ± 5% for 100 μg/mL treatment with CTS-TiO_2_-CO nanocomposite. These results altogether indicate that the nanoparticles were not highly toxic to the cells.

A step forward, we investigated the effect of CTS-TiO_2_-CO nanocomposite exposure in an in vivo system, zebrafish embryos. We could observe that after exposure to CTS-TiO_2_-CO nanocomposite, there was no developmental defect in the embryos. The embryos, as observed under a microscope, showed a similar morphology to that of untreated control embryos. No pericardial edema, tail bent, egg yolk edema, defect in the eye socket, length of the hatched embryo or change in thickness of the chorion layer was observed for the embryos (Figure 5c). The cumulative hatchability data (Figure 5d) showed that at 50 μg/mL and 100 μg/mL, the embryo viability was 88% and 82%, respectively, showing an acceptable biocompatibility. Moreover, there was no delayed hatching observed after treatment with the nanoparticles at any dose of exposure. These results altogether reveal that the synthesized nanoparticles are safe to use up to a dose of 100 μg/mL.

### 3.4. Textile Dye Remediation Using CTS-TiO_2_-CO Nanocomposite

**Figure 6:**
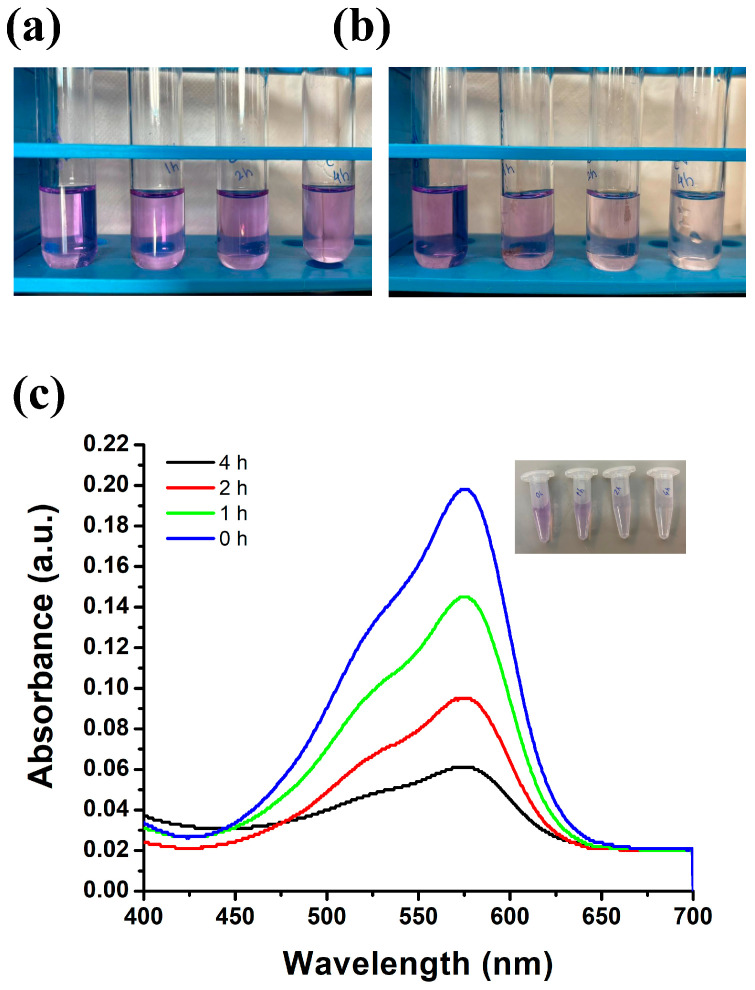
(**a**) The tubes containing crystal violet before exposure to sunlight and (**b**) after exposure to sunlight. (**c**) The absorption spectrum of crystal violet without sunlight exposure and after exposure to sunlight for 0 h, 1 h, 2 h, and 4 h. The inset tubes show the samples taken in small tubes.

Crystal violet is a dye that is commonly used in laboratories as well as in the textile industry. The effluent from those laboratories gets mixed with the water bodies and ultimately causes water pollution [62]. In an attempt to remediate these dyes and make them safe for the environment, we have designed the CTS-TiO_2_-CO nanocomposite. The dye remediation property was tested for crystal violet after adding the nanoparticles with the dye and exposing them to sunlight for different time intervals. The dye absorbance was used to monitor the degradation. Figure 6a shows the images of the tubes before sunlight exposure, and Figure 6b shows them after exposure. The dye degradation estimated using spectrophotometry is shown in Figure 6c.

The results show that the more the nanoparticle and dye mixture was exposed to sunlight, the more degradation was observed. The dye degradation percentage was also calculated using the absorption data. Absorption maximum (λ_max_) of crystal violet is at 582 nm.


$$\%\;CV\;degradation=\frac{\left(OD\;at\;0h\right)-(OD\;at\;x\;h)}{\left(OD\;at\;0h\right)}\times 100$$

The degradation of CV was 26.76%, 52.02%, and 69.19% after 1 h, 2 h, and 4 h exposure to sunlight. Photocatalytic degradation of crystal violet (CV) using CO-TiO_2_ nanoparticles initiates when UV or visible light excites electrons from the valence band to the conduction band, generating electron–hole pairs. The holes cause oxidation of water or hydroxide ions to produce •OH radicals, while electrons reduce dissolved oxygen to O_2_•^−^ radicals. These reactive oxygen species attack the molecule of CV through N-demethylation and oxidative cleavage of the conjugated chromophore, forming intermediates such as dimethylaminobenzophenone, followed by ring opening and mineralization into CO_2_ and H_2_O [63,64].

The green synthesis of TiO_2_ nanoparticles using *Calendula officinalis* extract presents a cost-effective, sustainable, and eco-friendly alternative to conventional methods. The presence of phytochemicals like saponins, tannins, carbohydrates, flavonoids, glycosides, reducing sugars, and coumarins contributes to the plant’s potential therapeutic properties, reduction of titanium ions and capping of nanoparticles, which ensures control, size, and stability. The chitosan coating enhanced the stability and biocompatibility. Furthermore, the different photophysical characterizations confirmed the stable formation of crystalline TiO_2_ nanoparticles with favourable size, peony flower shape, and highly stable surface charge. In the MTT assay, the nanoparticles exhibited dose-dependent cytotoxicity, a common and expected trend for nanomaterials. However, cell viability remained above 80% at 100 μg/mL, indicating minimal cytotoxic effects. The zebrafish embryo assay provided a valuable in vivo toxicity profile. Embryos exposed to up to 100 μg/mL of nanoparticles showed normal development, hatching, and survival rates, with no major morphological deformities. Collectively, the chitosan coating not only helps to improve the stability and dispersion of TiO_2_ nanoparticles in biological systems but also contributes to enhanced cellular interaction and reduced toxicity. The green-synthesized TiO_2_ nanoparticles exhibited excellent photocatalytic activity, effectively degrading crystal violet dye by 69.19% within 4 h. In a previous study, 225 mg of P-TiO_2_ nanoparticles were added to 75 mL of 5 mg/L of methylene blue and exposed to 1800 min of white light using a lamp simulating solar radiation after equilibrating for 30 min in the dark. The results showed complete degradation (100% reduction) in the dye within 30 min [65]. However, in our study, we obtained 69.19% reduction in CV in 4 h after treating with a much lower volume of the photocatalyst. This significant dye degradation under UV and sunlight exposure indicates the potential of these nanoparticles for environmental applications such as wastewater remediation, especially in textile industry effluents. The photocatalytic process relies on the generation of reactive oxygen species that break down harmful dye molecules into non-toxic byproducts like water and carbon dioxide. The use of *Calendula officinalis* extract in the synthesis provides a natural, non-toxic, and sustainable alternative to conventional chemical methods, making the entire process environmentally friendly and cost-effective.

## 4. Conclusions

Our study elucidates the synthesis of chitosan-entrapped TiO_2_ nanoparticles using *Calendula officinalis* extract. The nanocomposite was successfully synthesized, which had a good band gap to act as a semiconductor, essential for photocatalysis. The size of the composite was also in the nanometre scale, highlighting the advantages of a high surface area to volume ratio. The photocatalyst could degrade the textile dye, crystal violet, up to 69.19% within 4 h of exposure to sunlight, showing its efficacy as a photocatalyst. Moreover, being non-toxic and affordable, its practical value in real-world applications is enhanced. Our synthesized CTS-TiO_2_-CO nanocomposite has the potential to be designated as an efficient photocatalyst that can be used for wastewater remediation. However, the absorption capacity in the visible light region can be enhanced by appropriate doping with metals or non-metals, which can be explored in the future. Further research is needed to explore its reusability and capacity to degrade other dyes.

## Figures and Tables

**Table 1:** The FTIR peaks as obtained for the CO-TiO_2_ nanoparticles and CTS-TiO_2_-CO nanocomposite.

Peak (cm^−1^) for CTS-TiO_2_-CO Nanocomposite	Peak (cm^−1^) for CO-TiO_2_ Nanoparticles	Representing Bonds	Reference(s)
3126	3098	O-H stretching	[57]
2122	-	C-O mono-carbonyl	[57,58]
1526	1527	N-H bending of amide II	[59]
1419	1417	CH_2_ bending	[59]
1028	1025	C-O stretching	[59]
770, 613, 431	717, 584, 412	Ti-O-Ti	[60]

## Data Availability

The original contributions presented in this study are included in the article/Supplementary Materials. Further enquiries can be directed to the corresponding author.

## Supplementary Materials

The following supporting information can be downloaded at: https://www.mdpi.com/article/10.3390/polym18060745/s1, Figure S1: (a) The phytochemical analysis of *Calendula officinalis* extract; Tubes 1–13 represent the tests for Saponin, tannins, terpenoids and steroids, carbohydrates, flavonoids (Ferric chloride test), Flavonoids (alkaline test), glycosides, quinones, protein, reducing sugar, coumarins, alkaloids, and polysaccharides, respectively; (b) The GC-MS chromatogram of the CO extract, and the peaks for its components are shown in (c–g) representing 2-Formylhistamine, Imidazole-5-carboxylic acid, 2-amino-, 4-tert-Butylphenol, TMS derivative, d-Mannitol, 1-decylsulfonyl-, and Oxiraneoctanoic acid, 3-octyl-, cis-, respectively; Table S1: The chemical components present in the Calendula Officinalis flower extract, as revealed by GC-MS analysis.
