# Supplementary material for: Chitosan-Entrapped TiO2 Nanoparticles Synthesized Using Calendula officinalis Flower Extract—Photophysical Characterization, Biocompatibility, and Textile Dye Remediation
Source: Polymers (Basel). 2026 Mar 19;18(6):745. doi: 10.3390/polym18060745 (PMC13030242; doi:10.3390/polym18060745)
Supplement: Supplementary file 1 [file polymers-18-00745-s001.zip › Table S1.pdf]

Table S1. The chemical components present in the *Calendula Officinalis* flower extract, as revealed by GC-MS analysis.

S. No.	Compound Name	Molecular Formula	Molecular weight	Abundance (%)
1	2-Formylhistamine	$C_6H_9N_3O$	139	34.79
2	Imidazole-5-carboxylic acid, 2-amino-	$C_4H_5N_3O_2$	127	25.10
3	4-tert-Butylphenol, TMS derivative	$C_{13}H_{22}OSi$	222	12.20
4	d-Mannitol, 1-decylsulfonyl-	$C_{16}H_{34}O_7S$	370	12.66
5	Oxiraneoctanoic acid, 3-octyl-, cis-	$C_{18}H_{34}O_3$	298	15.25
