# Supplementary figures and images for: Chitosan-Entrapped TiO2 Nanoparticles Synthesized Using Calendula officinalis Flower Extract—Photophysical Characterization, Biocompatibility, and Textile Dye Remediation
Source: Polymers (Basel). 2026 Mar 19;18(6):745. doi: 10.3390/polym18060745 (PMC13030242; doi:10.3390/polym18060745)

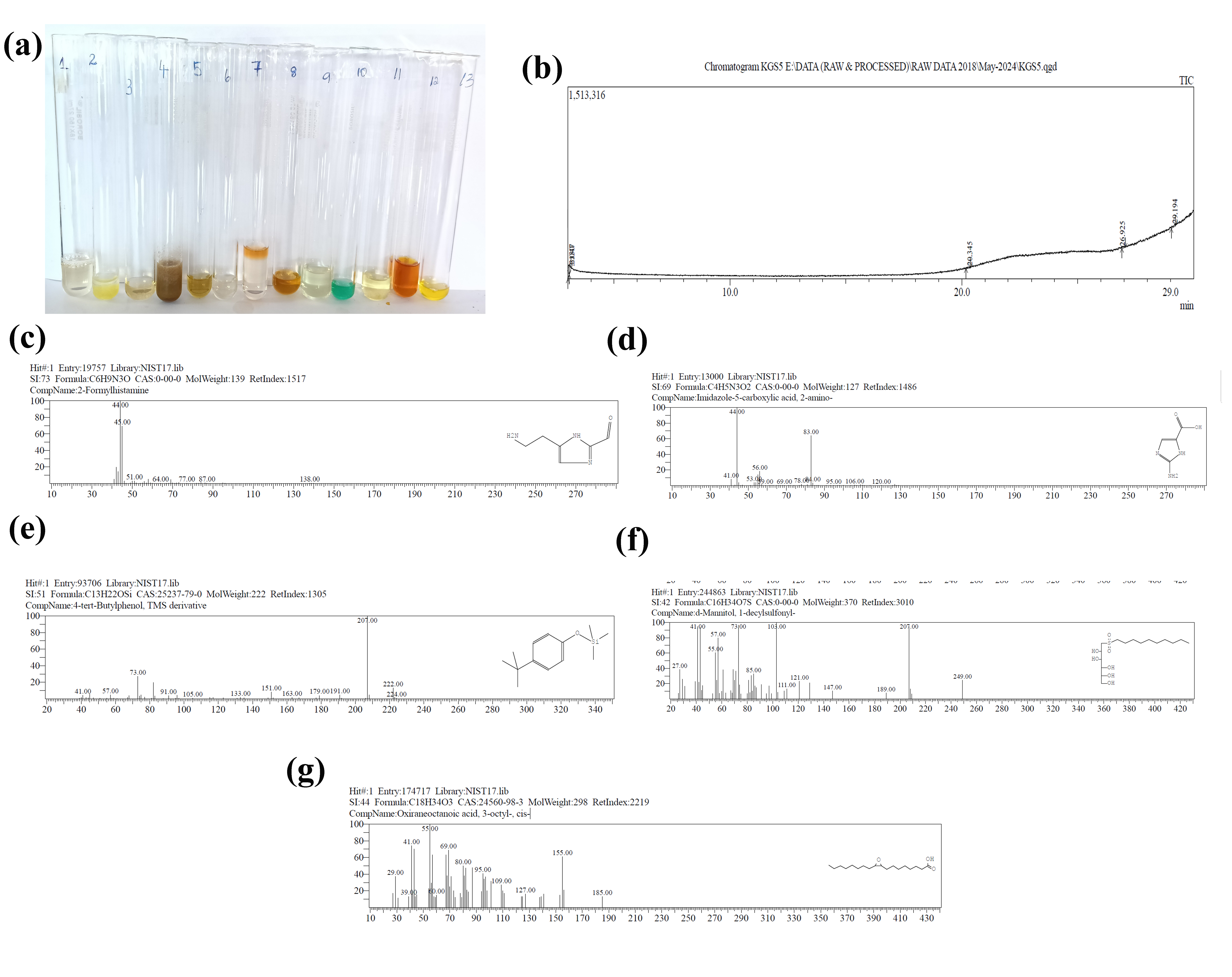

Supplement: Supplementary file 1 [file polymers-18-00745-s001.zip › Figure S1.jpg]
